# Variance Continuity for Lorenz Flows

**DOI:** 10.1007/s00023-020-00913-5

**Published:** 2020-05-02

**Authors:** Wael Bahsoun, Ian Melbourne, Marks Ruziboev

**Affiliations:** 1 Department of Mathematical Sciences, Loughborough University, Loughborough, LE11 3TU UK; 2 Mathematics Institute, University of Warwick, Coventry, CV4 7AL UK; 3 Mathematisch Instituut, Universiteit Leiden, Niels Bohrweg 1, 2333 CA Leiden, The Netherlands

**Keywords:** Primary 37A05, 37C10, 37E05

## Abstract

The classical Lorenz flow, and any flow which is close to it in the $C^2$-topology, satisfies a Central Limit Theorem (CLT). We prove that the variance in the CLT varies continuously.

## Introduction

In 1963, Lorenz [20] introduced the following system of equations:

1 $$\begin{aligned} {\left\{ \begin{array}{ll} \dot{x} = -10x+10y \\ \dot{y} = 28x-y-xz \\ \dot{z} = -\frac{8}{3}z+xy \end{array}\right. } \end{aligned}$$

as a simplified model for atmospheric convection. Numerical simulations performed by Lorenz showed that the above system exhibits sensitive dependence on initial conditions and has a non-periodic “strange” attractor. Since then, (1) became a basic example of a chaotic deterministic system that is notoriously difficult to analyse.

A rigorous mathematical framework of similar flows was initiated with the introduction of the so-called geometric Lorenz attractors in [1, 15]. The papers [24, 25] provided a computer-assisted proof that the classical Lorenz attractor in (1) is indeed a geometric Lorenz attractor. In particular, it is a singularly hyperbolic attractor [23], namely a nontrivial robustly transitive attracting invariant set containing a singularity (equilibrium point). Moreover, there is a distinguished Sinai–Ruelle–Bowen (SRB) ergodic probability measure; see for example [8]. Statistical limit laws, in particular the central limit theorem (CLT) for Hölder observables, were first obtained in [16] for the classical Lorenz attractor and were shown for general singular hyperbolic attractors in [5]. For further background and a complete list of references up to 2010, we refer the reader to the monograph of Araújo and Pacífico [7].

Let $X_\varepsilon :{\mathbb {R}}^3\times {\mathbb {R}}\rightarrow {\mathbb {R}}^3$, $\varepsilon \ge 0$, be a continuous family of $C^2$ flows on ${\mathbb {R}}^3$ admitting a geometric Lorenz attractor with singularities $x_\varepsilon$ and corresponding SRB measures $\mu _\varepsilon$. Precise definitions are given in Sect. 2.1; in particular, the framework includes the classical Lorenz attractor. By [2, 9], the flows $X_\varepsilon$ are statistically stable: for any continuous $\psi {:}{\mathbb {R}}^3\rightarrow {\mathbb {R}}$

$$\begin{aligned} \lim _{\varepsilon \rightarrow 0}\int \psi \, d\mu _{\varepsilon }=\int \psi \, \mathrm{d}\mu _0. \end{aligned}$$

The CLT in [5, 16] states that for fixed $\varepsilon \ge 0$ and $\psi {:}{\mathbb {R}}^3\rightarrow {\mathbb {R}}$ Hölder, there exists $\sigma ^2=\sigma ^2_{X_\varepsilon }(\psi )\ge 0$ such that

2 $$\begin{aligned} \frac{1}{\sqrt{t}}\left( \int _{0}^{t}\psi \circ X_{\varepsilon }(s)\, \mathrm{d}s -t\int \psi \, \mathrm{d}\mu _\varepsilon \right) {\overset{\text {law}}{\longrightarrow }} {\mathcal {N}}(0, \sigma ^2) \quad \text {as } t\rightarrow \infty . \end{aligned}$$

By [16, Section 4.3], $\sigma ^2$ is typically nonzero.

We prove continuity of the variance, namely that $\varepsilon \mapsto \sigma ^2_{X_\varepsilon }(\psi )$ is continuous. At the same time, we obtain estimates on the dependence of the variance on $\psi$. We now state the main result of the paper. Define $\Vert \psi \Vert =\int |\psi |\,\mathrm{d}\mu _0+|\psi (x_0)|$.

### Theorem 1.1

Let $\psi ,\,\psi '{:}{\mathbb {R}}^3\rightarrow {\mathbb {R}}$ be Hölder observables. Then,

- $$\lim _{\varepsilon \rightarrow 0}\sigma ^2_{X_\varepsilon }(\psi )=\sigma ^2_{X_0}(\psi )$$; and
- there exists a constant $C>0$ (depending only on the Hölder norms of $\psi$ and $\psi '$) such that
  $$\begin{aligned} |\sigma ^2_{X_0}(\psi )-\sigma ^2_{X_0}(\psi ')|\le C\Vert \psi -\psi '\Vert (1+|\log \Vert \psi -\psi '\Vert |). \end{aligned}$$

### Remark 1.2

In part (b), we obtain closeness of the variances provided the observables are close in $L^1$ with respect to both the SRB measure and the Dirac point mass at 0 (provided the individual Hölder norms are controlled). It is an easy consequence of the methods in this paper that the logarithmic factor can be removed if the norm $\Vert \;\Vert$ is replaced by the Hölder norm.

### Remark 1.3

All results in this paper go through without change for continuous families of $C^r$ flows, $r>1$. We take $r=2$ for notational convenience.

Our technique is based on first proving variance continuity for the corresponding family of Poincaré maps and then passing the result to the family of flows. The main difficulty in passing from maps to flows lies in the fact that the return time function to the Poincaré section is unbounded. A key step in overcoming this hurdle is to show that for any Hölder observable on ${\mathbb {R}}^3$ vanishing at the singularity, the induced observable for the Poincaré map is piecewise Hölder. Related results for various classes of discrete time dynamical systems can be found in [11, 13, 19] using somewhat different methods, but there are no previous results for Lorenz attractors.

The paper is organised as follows. In Sect. 2, we recall the basic set-up and notation associated with (families of) geometric Lorenz attractors. In Sect. 3, we show how to normalise the families of flows to obtain simplified coordinates for the proofs. Section 4 contains properties of one-dimensional Lorenz maps. Section 5 studies the family of Poincaré maps. It starts by showing that the family of maps admit a uniform rate of correlations decay for piecewise Hölder functions, using suitable anisotropic norms. We then use the Green–Kubo formula to show continuity of the variance for the family of Poincaré maps. In Sect. 6, we prove a version of Theorem 1.1 for normalised families and use this to prove Theorem 1.1.

## Geometric Lorenz Attractors

In this section, we recall the basic set-up and notation associated with (families of) geometric Lorenz attractors. In Sects. 2.1 and 2.2, we recall the class of (families of) geometric Lorenz attractors considered in the paper.

We begin with some notational preliminaries. Let $U\subset {\mathbb {R}}^m$ be open. Fix $\alpha \in (0,1)$ and recall that $f{:}U\rightarrow {\mathbb {R}}^n$ is $C^\alpha$ if there exists $C>0$ such that $|f_j(x)-f_j(y)|\le C|x-y|^\alpha$ for all $x,y\in U$ and all $j=1,\dots ,n$. (Here, $|x|=\sqrt{x_1^2+\dots +x_m^2}$ denotes the Euclidean norm on ${\mathbb {R}}^m$.) Let $H_{\alpha }(f)$ be the least such constant *C* and define the Hölder norm $\Vert f\Vert _{\alpha }=|f|_\infty +H_\alpha (f)$. Then, *f* is $C^{1+\alpha }$ if $Df{:}U\rightarrow {\mathbb {R}}^{n\times m}$ is $C^\alpha$ and we set $\Vert f\Vert _{1+\alpha }=|f|_\infty +\Vert Df\Vert _{\alpha }$. A family $f_\varepsilon$ of $C^{1+\alpha }$ maps, $\varepsilon \ge 0$, is said to be continuous if $\lim _{\varepsilon \rightarrow \varepsilon _0}\Vert f_\varepsilon -f_{\varepsilon _0}\Vert _{1+\alpha }=0$. Similarly, we speak of continuous families of $C^2$ flows, $C^{1+\alpha }$ diffeomorphisms, and so on. In the case of Lorenz flows, we are particularly interested in families of $C^2$ flows on ${\mathbb {R}}^3$ restricted to an open-bounded region *U* of phase space; for convenience, we suppress mentioning the subset *U*.

### Definition of Geometric Lorenz Attractors

There are various notions of geometric Lorenz attractor in the literature depending on the properties being analysed. Roughly speaking, we take a geometric Lorenz attractor to be a singular hyperbolic attractor for a vector field on ${\mathbb {R}}^3$ with a single singularity $x_0$ and a connected global cross section with a $C^{1+\alpha }$ stable foliation. As promised, we now give a precise description.

Let $G{:}{\mathbb {R}}^3\rightarrow {\mathbb {R}}^3$ be a $C^2$ vector field satisfying $G(x_0)=0$ and let *X* be the associated flow. We suppose that the differential $DG(x_0)$ at the singularity has three real eigenvalues $\lambda _2<\lambda _3<0<\lambda _1$ satisfying $\lambda _1+\lambda _3>0$ (Lorenz-like singularity).

Let $\Sigma$ be a two-dimensional rectangular cross section transverse to the flow chosen in a neighbourhood of the singularity $x_0$, and let $\Gamma$ be the intersection of $\Sigma$ with the local stable manifold of $x_0$. We suppose that there exists a well-defined Poincaré map $F{:}\Sigma {\setminus }\Gamma \rightarrow \Sigma$. Moreover, we assume that the underlying flow is singular hyperbolic [23]. It follows [4, Theorem 4.2] that a neighbourhood of the attractor is foliated by one-dimensional $C^2$ stable leaves. We assume *q*-bunching for some $q>1$ in [4, condition (4.2)]. By [4, Theorem 4.12 and Remark 4.13(b)], it follows that the stable foliation for the flow is $C^q$.

The foliation by stable leaves for the flow naturally induces (see for example [5, Section 3.1]) a $C^q$ ($q>1$) foliation inside $\Sigma$ of a neighbourhood of the attractor intersected with $\Sigma$ by one-dimensional $C^2$ stable leaves for the Poincaré map *F*. We denote this stable foliation for *F* by ${\mathcal F}$.

The stable leaves for the flow are exponentially contracting [4, Theorem 4.2(a)(3)], and this property is inherited by the stable leaves for *F*. This means that there exists $\rho \in (0,1)$, $K>0$ such that

3 $$\begin{aligned} |F^n \xi _1- F^n \xi _2|\le K\rho ^n|\xi _1- \xi _2|, \end{aligned}$$

for $\xi _1,\xi _2$ in the same stable leaf in ${\mathcal F}$ and $n\ge 1$.

Let $I\subset \Sigma$ be a smoothly ($C^\infty$) embedded one-dimensional subspace transverse to the stable foliation, and let $T{:}I\rightarrow I$ be the one-dimensional map obtained from *F* by quotienting along stable leaves. Let $\xi _0$ be the intersection of *I* with $\Gamma$.

#### Proposition 2.1

*T* is a Lorenz-like expanding map. That is, *T* is monotone (without loss we take *T* to be increasing) and piecewise $C^{1+\alpha }$ on $I{\setminus }\{\xi _0\}$ for some $\alpha \in (0,1)$ with a singularity at $\xi _0$ and one-sided limits $T(\xi _0^+)<0$ and $T(\xi _0^-)>0$. Also, *T* is uniformly expanding: there are constants $c>0$ and $\theta >1$ such that $(T^n)'(x)\ge c\theta ^{n}$ for all $n\ge 1$, whenever $x\notin \bigcup _{j=0}^{n-1}T^{-j}(\xi _0)$.

#### Proof

The map *F* is piecewise $C^{1+\alpha }$, and the foliation by stable leaves is $C^{1+\alpha }$, so *T* is piecewise $C^{1+\alpha }$ on $I{\setminus }\{\xi _0\}$. Uniform expansion follows from [5, Theorem 4.3]. The remaining properties are immediate. $\square$

The final part of the definition of geometric Lorenz attractor is that the one-dimensional map *T* is transitive on the interval $[T(\xi _0^+),T(\xi _0^-)]$. It is then standard [2, 5, 8, 17] that *T* has a unique absolutely continuous invariant probability measure (acip) ${\bar{\mu }}$ leading to a unique SRB measure $\mu$ for the geometric Lorenz attractor containing $x_0$. The basin of $\mu$ has full Lebesgue measure in a neighbourhood of the attractor.

#### Remark 2.2

The classical Lorenz attractor for the system of equations (1) (and for nearby equations) is an example of a geometric Lorenz attractor as defined above. Except for *q*-bunching, the assumptions above are verified in [25]. The *q*-bunching condition is verified in [6, Lemma 2.2]. (By [4, Section 5], the optimal value of *q* lies between 1.278 and 1.705; hence, we have $C^{1+\alpha }$ regularity for the stable foliation as in [9] but not $C^2$ regularity as in [2].)

### Families of Geometric Lorenz Attractors

Let $X_\varepsilon$, $\varepsilon \ge 0$, be a continuous family of $C^2$ flows on ${\mathbb {R}}^3$ admitting a geometric Lorenz attractor as in Sect. 2.1 with singularity $x_\varepsilon$. The constants *K* and $\rho$ in (3) derive from the singular hyperbolic structure which varies continuously under $C^1$ perturbations. Hence, *K* and $\rho$ can be chosen independent of $\varepsilon$.

Making an initial $C^2$ change of coordinates (varying continuously in $\varepsilon$), we can suppose without loss that $x_\varepsilon \equiv 0$ and that $\Sigma$, $\Gamma$ and *I* are given by $\Sigma =\left\{ (x, y, 1){:}-1\le x\le 1,\,-\frac{1}{2}\le y \le \frac{1}{2} \right\}$, $\Gamma =\{(0, y, 1){:} -\frac{1}{2} \le y \le \frac{1}{2}\}$ and $I=\{(x, 0, 1){:} -\frac{1}{2} \le x \le \frac{1}{2}\}\cong [-\frac{1}{2},\frac{1}{2}]$ for all $\varepsilon$. Throughout the paper, when we speak of a continuous family of $C^2$ flows admitting geometric Lorenz attractors, we assume that this initial change of coordinates has been performed.

Define the Poincaré return time to $\Sigma$,

$$\begin{aligned} \tau _\varepsilon {:}\Sigma \rightarrow (0,\infty ), \qquad \tau _\varepsilon (\xi )=\inf \{t>0{:}X_\varepsilon (\xi ,t)\in \Sigma \}. \end{aligned}$$

#### Proposition 2.3

The return time $\tau _\varepsilon {:}\Sigma {\setminus }\Gamma \rightarrow \Sigma$ is given by $\tau _\varepsilon (x, y)=-\frac{1}{\lambda _{1,\varepsilon }} \log |x|+\tau _{2,\varepsilon }(x,y)$ where $\tau _{2,\varepsilon }$ is a continuous family of $C^2$ functions.

#### Proof

Applying the Hartman–Grobman theorem for fixed $\varepsilon$, we can linearise $X_\varepsilon$ in a neighbourhood of 0 so that the linearised flow is given by $(x,y,z)\mapsto (e^{\lambda _{1,\varepsilon } t}x,e^{\lambda _{2,\varepsilon } t}y,e^{\lambda _{3,\varepsilon } t}z)$. The time of flight in this neighbourhood is readily calculated in these coordinates to be $-\frac{1}{\lambda _{1,\varepsilon }} \log |x|$ for $x\in I$ and the same formula holds in the original coordinates. The remaining flight time $\tau _{2,\varepsilon }$ is a first hit time for the $C^2$ flow away from the singularity at 0 and hence is $C^2$. Since $X_\varepsilon$ is a continuous family of $C^2$ flows, it follows that $\tau _{2,\varepsilon }$ is a continuous family of $C^2$ functions. $\square$

#### Theorem 2.4

Let $X_\varepsilon$ be a continuous family of $C^2$ flows on ${\mathbb {R}}^3$ admitting a geometric Lorenz attractor. Then, there exists $\alpha >0$ such that the one-dimensional maps $T_\varepsilon {:}I\rightarrow I$ form a continuous family of piecewise $C^{1+\alpha }$ maps.

#### Proof

Recall that $T_\varepsilon$ is obtained from the continuous family of piecewise $C^{1+\alpha }$ maps $F_\varepsilon$ by quotienting along the stable foliation. Our assumption of *q*-bunching ($q>1$) yields continuous families of $C^q$ stable foliations [12]. Hence, $T_\varepsilon$ is a continuous family of piecewise $C^{1+\alpha }$ maps. $\square$

## Normalised Geometric Lorenz Attractors

Let $X_\varepsilon$, $\varepsilon \ge 0$, be a continuous family of $C^2$ flows on ${\mathbb {R}}^3$ admitting a geometric Lorenz attractor. In this section, we show how to normalise the families of flows to obtain simplified coordinates for carrying out the proofs.

Assume that the preliminary $C^2$ change of coordinates in Sect. 2.2 has been performed. Let $F_\varepsilon {:}\Sigma \rightarrow \Sigma$ and $T_\varepsilon {:}I\rightarrow I$ be the associated families of Poincaré maps and one-dimensional piecewise expanding maps. Also, define ${\widetilde{\Sigma }}=\left\{ (x, y, 1){:}-\frac{1}{2}\le x, y \le \frac{1}{2} \right\}$.

### Proposition 3.1

There exists a continuous family of $C^{1+\alpha }$ diffeomorphisms $\omega _\varepsilon {:}\Sigma \rightarrow \Sigma$ such that

- $\omega _\varepsilon$ restricts to the identity on *I*; and
- vertical lines in ${\widetilde{\Sigma }}$ are transformed under $\omega _\varepsilon$ into stable leaves for $F_\varepsilon {:}\Sigma \rightarrow \Sigma$.

### Proof

For $\varepsilon$ fixed, this follows by definition of the smoothness of the stable foliation for $F_\varepsilon$. (An explicit formula is given in [4, Lemma 4.9] where *Y* and $\chi$ should be replaced by *I* and $\omega _\varepsilon$, and the embedding is the identity.) Again, we obtain continuous families of $C^{1+\alpha }$ diffeomorphisms by [12]. $\square$

The change of coordinates $\omega _\varepsilon$ for the Poincaré map $F_\varepsilon$ extends to a change of coordinates $\phi _\varepsilon$ for the flow $X_\varepsilon$. The extension is standard and essentially unique, though heavy on notation.

First, define the transformed Poincaré map and return time

$$\begin{aligned} {\tilde{F}}_\varepsilon =\omega _\varepsilon ^{-1}\circ F_\varepsilon \circ \omega _\varepsilon {:}{\widetilde{\Sigma }}\rightarrow {\widetilde{\Sigma }}, \qquad {{\tilde{\tau }}}_\varepsilon =\tau _\varepsilon \circ \omega _\varepsilon {:}{\widetilde{\Sigma }}\rightarrow (0,\infty ). \end{aligned}$$

The set $U_\varepsilon =\{(X_\varepsilon (\xi ,t){:}\xi \in \Sigma ,\,0\le t\le \tau _\varepsilon (\xi )\}$ defines a neighbourhood of the Lorenz attractor for $X_\varepsilon$. Note that $X_\varepsilon (\xi ,\tau _\varepsilon (\xi ))=F_\varepsilon \xi$ for $\xi \in \Sigma$. Define the transformed flow ${\tilde{X}}_\varepsilon {:}U_\varepsilon \times [0,\infty )\rightarrow U_\varepsilon$ given by ${\tilde{X}}_\varepsilon (x,t)=X_\varepsilon (x,t)$ subject to the identifications ${\tilde{X}}_\varepsilon (\xi ,{{\tilde{\tau }}}_\varepsilon (\xi ))=\tilde{F}_\varepsilon \xi$ for $\xi \in {\widetilde{\Sigma }}$.

Finally, define $\phi _\varepsilon {:}U_\varepsilon \rightarrow U_\varepsilon$ by $\phi _\varepsilon (x)=X_\varepsilon (\omega _\varepsilon (\xi ),t)$ for $x= X_\varepsilon (\xi ,t)\in U_\varepsilon$, where $\xi \in \Sigma$ and $0\le t\le {{\tilde{\tau }}}_\varepsilon (x)$. It follows from the definitions that $\phi _\varepsilon |_{\Sigma _\varepsilon }\equiv \omega _\varepsilon$ and $\tilde{X}_\varepsilon (x,t)=\phi _\varepsilon ^{-1}\circ X_\varepsilon (\phi _\varepsilon (x),t)$. Hence, $\phi _\varepsilon$ is the desired change of coordinates.

### Corollary 3.2

The changes of coordinates $\phi _\varepsilon {:}U\rightarrow U$ are continuous families of $C^{1+\alpha }$ diffeomorphisms.

### Proof

By assumption, $X_\varepsilon$ is a continuous family of $C^2$ flows. Hence, the result follows from Proposition 3.1. $\square$

### Lemma 3.3

The transformed flow ${\tilde{X}}_\varepsilon (x,t)=\phi _\varepsilon ^{-1}\circ X_\varepsilon (\phi _\varepsilon (x),t)$ satisfies:

(i) The Poincaré map ${\tilde{F}}_\varepsilon {:}{\widetilde{\Sigma }}{\setminus }\Gamma \rightarrow {\widetilde{\Sigma }}$ is a continuous family of piecewise $C^{1+\alpha }$ diffeomorphisms and has the form
  $$\begin{aligned} {\tilde{F}}_\varepsilon (x,y)=(T_\varepsilon x,g_\varepsilon (x,y)), \end{aligned}$$
  where $T_\varepsilon {:}I\rightarrow I$ is the family of Lorenz-like expanding maps in Theorem 2.4(a).
(ii) $|{\tilde{F}}_\varepsilon ^n\xi _1- {\tilde{F}}_\varepsilon ^n\xi _2|\le K\rho ^n|\xi _1- \xi _2|$ for all $\xi _1=(x,y_1),\,\xi _2=(x,y_2)$ and $n\ge 1,$
(iii) The return time ${{\tilde{\tau }}}_\varepsilon {:}{\widetilde{\Sigma }}{\setminus }\Gamma \rightarrow (0,\infty )$ is given by ${{\tilde{\tau }}}_\varepsilon (x, y)=-\frac{1}{\lambda _{1,\varepsilon }} \log |x|+{{\tilde{\tau }}}_{2,\varepsilon }(x,y)$ where ${{\tilde{\tau }}}_{2,\varepsilon }$ is a continuous family of $C^{1+\alpha }$ functions.
(iv) The SRB measures ${{\tilde{\mu }}}_\varepsilon$ for ${\tilde{X}}_\varepsilon$ are given by ${{\tilde{\mu }}}_\varepsilon ={\phi _{\varepsilon }^{-1}}\!_*\mu _\varepsilon$ and are statistically stable.

### Proof

(i) By Proposition 3.1(b), $\tilde{F}_\varepsilon =\omega _\varepsilon ^{-1}\circ F_\varepsilon \circ \omega _\varepsilon$ has the form ${\tilde{F}}_\varepsilon (x,y)=(T_\varepsilon x,g_\varepsilon (x,y))$. By Proposition 3.1(a), the maps $T_\varepsilon {:}I\rightarrow I$ are unchanged by this change of coordinates. Also, $\omega _\varepsilon$ is a continuous family of $C^{1+\alpha }$ diffeomorphisms and $F_\varepsilon$ is a continuous family of piecewise $C^{1+\alpha }$ diffeomorphisms, so ${\tilde{F}}_\varepsilon$ a continuous family of piecewise $C^{1+\alpha }$ diffeomorphisms.
(ii) This is the uniform contraction condition (3) in the new coordinates.
(iii) By Proposition 2.3 and Proposition 3.1, ${{\tilde{\tau }}}_\varepsilon =\tau _\varepsilon \circ \omega _\varepsilon =-\frac{1}{\lambda _{1,\varepsilon }} \log |x|+\tau _{2,\varepsilon }\circ \omega _\varepsilon$ where ${{\tilde{\tau }}}_{2,\varepsilon }=\tau _{2,\varepsilon }\circ \omega _\varepsilon$ is a continuous family of $C^{1+\alpha }$ functions.
(iv) By Proposition 3.1(a), the acip ${\bar{\mu }}_\varepsilon$ for $T_\varepsilon$ is unchanged by the change of coordinates and hence remains absolutely continuous. Using this and the construction of the SRB measure (see for example [2, 8] for the standard construction of $\mu _\varepsilon$ from ${\bar{\mu }}_\varepsilon$), we obtain that ${{\tilde{\mu }}}_\varepsilon ={\phi _\varepsilon ^{-1}}\!_*\mu _\varepsilon$ is the SRB measure for ${\tilde{X}}_\varepsilon$. Moreover, strong statistical stability [2, 9, 14] of the acips ${\bar{\mu }}_\varepsilon$ on *I* is preserved and hence the SRB measures ${{\tilde{\mu }}}_\varepsilon$ remain statistically stable.

$$\square $$

In the following sections, we prove Theorem 1.1 for normalised families of geometric Lorenz attractors. At the end of Sect. 6, we show how results for normalised families imply Theorem 1.1.

## The Family of One-Dimensional Maps

In this section, we recall some functional–analytic properties associated with the family of one-dimensional Lorenz maps $T_\varepsilon$. For $p\ge 1$, we say $f{:}I \rightarrow {\mathbb {R}}$ is of (universally) bounded *p*-variation if

$$\begin{aligned} V_p(f)= \sup _{-\frac{1}{2}= x_0<\dots<x_n= \frac{1}{2}} \left( \sum _{i=1}^{n}\left| f(x_i)-f(x_{i-1})\right| ^p \right) ^{1/p} <\infty . \end{aligned}$$

We take $$p\ge \frac{1}{\alpha }$$.

Let $S_\rho (x)=\{y\in I{:} |x-y|<\rho \}$. For $f:I\rightarrow {\mathbb {R}}$, define

$$\begin{aligned} {{\,\mathrm{osc}\,}}(f, \rho , x)= {{\,\mathrm{ess sup}\,}}\{|f(y_1)-f(y_2)|: y_1, y_2\in S_\rho (x)\}, \end{aligned}$$

and

$$\begin{aligned} {{\,\mathrm{osc}\,}}_1(f, \rho )=\Vert {{\,\mathrm{osc}\,}}(f, \rho , x)\Vert _1, \end{aligned}$$

where the essential supremum is taken with respect to Lebesgue measure on $I\times I$ and $\Vert \cdot \Vert _1$ is the $L^1$- norm with respect to Lebesgue measure on *I*. Fix $\rho _0>0$ and let $BV_{1, 1/p}\subset L^1$ be the Banach space equipped with the norm

$$\begin{aligned} \Vert f\Vert _{1, 1/p}=V_{1, 1/p}(f)+\Vert f\Vert _1, \quad \text {where }\quad V_{1, 1/p}(f)=\sup _{0<\rho \le \rho _0}\frac{{{\,\mathrm{osc}\,}}_1(f, \rho )}{\rho ^{1/p}}. \end{aligned}$$

(The space $BV_{1, 1/p}$ does not depend on $\rho _0$.) The fact that $BV_{1, 1/p}$ is a Banach space is proved in [17]. Moreover, it is proved in [17] that $BV_{1, 1/p}$ is embedded in $L^\infty$ and compactly embedded in $L^1$. In addition, [17] shows that

4 $$\begin{aligned} V_{1,1/p}(f)\le 2^{1/p}V_p(f). \end{aligned}$$

We recall results from the literature that we use later in Sects. 5 and 6of the paper. Recall from [17] that $T_\varepsilon$ admits a unique acip ${\bar{\mu }}_\varepsilon$ for each $\varepsilon \ge 0$. Let $h_\varepsilon$ denote the density for ${\bar{\mu }}_\varepsilon$. Let $P_{\varepsilon }:L^1(I)\rightarrow L^1(I)$ denote the transfer operator (Perron–Frobenius) associated with $T_\varepsilon$ (so $\int P_\varepsilon f\,g\,d{{\,\mathrm{Leb}\,}}=\int f\,g\circ T_\varepsilon \,d{{\,\mathrm{Leb}\,}}$ for $f\in L^1(I)$, $g\in L^\infty (I)$).

### Proposition 4.1

There exist $C>0$ and $\Lambda \in (0,1)$ such that

- $$||h_\varepsilon ||_\infty <C$$, and
- $$\Vert P^n_\varepsilon f-h_\varepsilon \int f\,d{{\,\mathrm{Leb}\,}}\Vert _{1,1/p}\le C\Lambda ^n||f||_{1, 1/p}$$

for all $$n\ge 1$$, $$\varepsilon \ge 0$$, $$f\in BV_{1,1/p}$$.

### Proof

By [9, Lemma 3.3], there exist $A_1, A_2>0$, $0<\kappa <1$, such that for all $n\ge 1$, $f\in BV_{1, 1/p}$,

$$\begin{aligned} ||P^n_\varepsilon f||_{1,1/p}\le A_1\kappa ^n||f||_{1, 1/p}+A_2||f||_1. \end{aligned}$$

Taking $f=h_\varepsilon$ and letting $n\rightarrow \infty$, we obtain $\Vert h_\varepsilon \Vert _{1,1/p}\le A_2$ and part (a) follows. Moreover, it was proved in [9] that

$$\begin{aligned} \lim _{\varepsilon \rightarrow 0}\sup _{||f||_{1,1/p}\le 1}||(P_\varepsilon -P_0)f||_1=0. \end{aligned}$$

Thus, the Keller–Liverani stability result [18] implies that $P_\varepsilon$ has a uniform (in $\varepsilon$) spectral gap on $BV_{1,1/p}$. This proves part (b). $\square$

## Variance Continuity for the Poincaré Maps

In this section, we prove the analogue of Theorem 1.1 at the level of the Poincaré maps $F_\varepsilon$. Throughout, we work with normalised families as in Sect. 3.

It is well known [8] that $F_\varepsilon$ admits a unique SRB measure $\mu _{F_\varepsilon }$ for each $\varepsilon \ge 0$. Moreover, for any continuous $\psi :\Sigma \rightarrow {\mathbb {R}}$ we have

$$\begin{aligned} \lim _{\varepsilon \rightarrow 0}\int \psi \, \mathrm{d}\mu _{F_\varepsilon }=\int \psi \, \mathrm{d}\mu _{F_0}; \end{aligned}$$

i.e. the family $F_\varepsilon$ is statistically stable [2, 9, 14]. We require the following strengthening of this property. In general, we say that $\Psi :\Sigma \rightarrow {\mathbb {R}}$ is piecewise continuous if it is uniformly continuous on the connected components of $\Sigma {\setminus }\Gamma$. Similarly, $\Psi$ is piecewise Hölder if it is uniformly Hölder on the connected components of $\Sigma {\setminus }\Gamma$.

### Proposition 5.1

Suppose that $\Psi {:}\Sigma \rightarrow {\mathbb {R}}$ is piecewise continuous and fix $n\ge 0$. Then, $\lim _{\varepsilon \rightarrow 0}\int \Psi \cdot (\Psi \circ F_0^n)\,\mathrm{d}\mu _{F_\varepsilon }=\int \Psi \cdot (\Psi \circ F_0^n)\,\mathrm{d}\mu _{F_0}$.

### Proof

If $\Psi \cdot (\Psi \circ F_0^n)$ were continuous, this would be immediate from the statement of Proposition 3.3 in [2]. The proof in [2] already accounts for trajectories that visit $\Gamma$ in finitely many steps, and it is easily checked that the same arguments apply here. $\square$

For fixed $\varepsilon \ge 0$, the CLT holds (see for instance [21]); i.e. for $\Psi {:}\Sigma \rightarrow {\mathbb {R}}$ piecewise Hölder there exists $\sigma ^2=\sigma ^2_{F_\varepsilon }(\Psi )$ such that

$$\begin{aligned} \frac{1}{\sqrt{n}}\left( \sum _{i=0}^{n-1}\Psi \circ F_\varepsilon ^i-n\int \Psi \, \mathrm{d}\mu _{F_\varepsilon }\right) {\overset{\text {law}}{\longrightarrow }}\mathcal N(0, \sigma ^2) \quad \text {as } n\rightarrow \infty . \end{aligned}$$

The variance satisfies the Green–Kubo formula

5 $$\begin{aligned} \sigma ^2=\int {\hat{\Psi }}_\varepsilon ^2\,\mathrm{d}\mu _{F_\varepsilon }+2\sum _{n=1}^{\infty }\int {\hat{\Psi }}_\varepsilon \cdot ({\hat{\Psi }}_\varepsilon \circ F^n_{\varepsilon })\, \mathrm{d}\mu _{F_\varepsilon }, \end{aligned}$$

where $${\hat{\Psi }}_\varepsilon =\Psi _\varepsilon -\int \Psi _\varepsilon \, \mathrm{d}\mu _{F_\varepsilon }$$.

### Uniform Decay of Correlations

Let $\alpha \in (0,1]$ and $p\ge \frac{1}{\alpha }$. Let $\Psi {:}\Sigma \rightarrow {\mathbb {R}}$ be piecewise Hölder with exponent $\alpha$. Set

$$\begin{aligned} ||\Psi ||_{\alpha ,s}=H_{\alpha ,s}(\Psi )+||\Psi ||_{\infty }, \qquad H_{\alpha ,s}(\Psi )=\sup _{\underset{y_1\not =y_2}{x,y_1,y_2\in I}}\frac{|\Psi (x,y_2)-\Psi (x,y_1)|}{|y_2-y_1|^\alpha }. \end{aligned}$$

For $-\frac{1}{2}=x_0< \dots < x_n=\frac{1}{2}$ and $y_i\in I$, $1\le i\le n$, we define

$$\begin{aligned} {\hat{V}}_p(\Psi ;(x_0,\dots ,x_n); (y_1,\dots ,y_n))= \left( \sum _{1\le i\le n}|\Psi (x_{i-1},y_i)-\Psi (x_{i}, y_i)|^p\right) ^{1/p}, \end{aligned}$$

and

$$\begin{aligned} {\hat{V}}_p(\Psi )=\sup {\hat{V}}_p(\Psi ;(x_0,\dots ,x_n); (y_1,\dots ,y_n)), \end{aligned}$$

where the supremum is taken over all finite partitions of *I* and all choices of $y_i\in I$. Finally, we define

$$\begin{aligned} (\Pi \Psi )(x)=\int _I\Psi (x,y)\,dy. \end{aligned}$$

We state and prove the following theorem about uniform (in $\varepsilon$) decay of correlations. Define

$$\begin{aligned} D_{\Psi }=||\Pi \Psi ||_{1,1/p}+{\hat{V}}_p(\Psi )+||\Psi ||_{\alpha ,s}. \end{aligned}$$

Note that $D_\Psi$ is finite.

#### Theorem 5.2

Assume that there exists $K>0$ such that

6 $$\begin{aligned} ||\Pi (\Psi \circ F_\varepsilon ^j)||_{1,1/p}+{\hat{V}}_p(\Psi \circ F_\varepsilon ^j)\le D_\Psi K^j \end{aligned}$$

for all $j\ge 1$, $\varepsilon \ge 0$. Then, there exist $C>0$ and $\theta \in (0,1)$ such that

$$\begin{aligned} \begin{aligned}&\left| \int \Psi \cdot (\Psi \circ F_\varepsilon ^n)\, \mathrm{d}\mu _{F_\varepsilon }-\Big (\int \Psi \, \mathrm{d}\mu _{F_\varepsilon }\Big )^2\right|&\le C D_{\Psi }||\Psi ||_{\alpha ,s} \theta ^n \end{aligned} \end{aligned}$$

for all $$n\ge 1$$, $$\varepsilon \ge 0$$.

#### Proof

The proof follows from [3, Theorem 3]. The fact that *C* and $\theta$ do not depend on $\varepsilon$ follows from the uniformity of the constants in Proposition 4.1(b) and the uniformity of the contraction on vertical fibres. $\square$

We now verify assumption (6) in Theorem 5.2.

#### Lemma 5.3

$$V_{1,1/p}(\Pi \Psi )\le 2^{1/p}{\hat{V}}_p(\Psi )$$.

#### Proof

Fix $y\in I$ and let $x_0<\dots <x_n$ be a partition of *I*. We have

$$\begin{aligned} \begin{aligned} \sum _{1\le i\le n}|\Pi \Psi (x_{i-1})-\Pi \Psi (x_{i})|^p&= \sum _{1\le i\le n}\Big |\int (\Psi (x_{i-1},y)-\Psi (x_{i}, y))\,dy\Big |^p \\&\le \sum _{1\le i\le n}\int |\Psi (x_{i-1},y)-\Psi (x_{i}, y)|^p \,dy \le {\hat{V}}_p(\Psi )^p. \end{aligned} \end{aligned}$$

Combining this with (4), we have $V_{1,1/p}(\Pi \Psi )\le 2^{1/p}V_p(\Pi \Psi )\le 2^{1/p}{\hat{V}}_p(\Psi )$ as required. $\square$

Recall that $F_\varepsilon$ can be written in coordinates as $F_\varepsilon (x,y)=(T_\varepsilon x,g_\varepsilon (x,y))$.

#### Lemma 5.4

Let $$M=4(1+\sup _\varepsilon \Vert g_\varepsilon \Vert _{C^1}^{\alpha })$$. Then,

$$\begin{aligned} {\hat{V}}_p(\Psi \circ F_\varepsilon ^j)\le (2^j-1)M||\Psi ||_{\alpha ,s}+2^j{\hat{V}}_p(\Psi ) \quad \text {for all } j\ge 1, \varepsilon \ge 0. \end{aligned}$$

#### Proof

We suppress the dependence on $\varepsilon$. Fix $-\frac{1}{2}=x_0\le x_1\le \cdots \le x_n=\frac{1}{2}$ and $y_i\in I$, $1\le i\le n$. Then,

$$\begin{aligned}&{\hat{V}}_p(\Psi \circ F;(x_0,\dots ,x_n); (y_1,\dots ,y_n))^p =\sum _{1\le i\le n}|\Psi \circ F(x_{i-1},y_i)-\Psi \circ F(x_{i}, y_i)|^p\\&\quad \le 2^{p-1}\sum _{1\le i\le n}H_{\alpha ,s}(\Psi )^p|g(x_{i-1},y_i) -g(x_{i},y_i)|^{\alpha p}\\&\qquad +2^{p-1}\sum _{1\le i\le n}|\Psi (T x_{i-1},g(x_{i-1},y_i)) -\Psi (T x_{i},g(x_{i-1},y_i))|^p\\&\quad \le 2^{p-1}H_{\alpha ,s}(\Psi )^p {\Vert g\Vert }_{C^1}^{\alpha p} \\&\qquad + 2^{p-1}\sum _{1\le i\le n}|\Psi (T x_{i-1},g(x_{i-1},y_i))-\Psi (T x_{i},g(x_{i-1},y_i))|^p. \end{aligned}$$

Let $x_0,\dots , x_k\in [-\frac{1}{2},0)$ and $x_{k+1}\notin [-\frac{1}{2},0)$. Since $T|_{[-\frac{1}{2},0)}$ is continuous and increasing, we get

$$\begin{aligned} \sum _{1\le i\le k}|\Psi (T x_{i-1},g(x_{i-1},y_i))-\Psi (T x_{i},g(x_{i-1},y_i))|^p\le {\hat{V}}_p(\Psi )^p. \end{aligned}$$

A similar estimate holds for $x_{k+1},\dots , x_n\in (0,\frac{1}{2}]$. Moreover,

$$\begin{aligned} |\Psi (T x_{k},g(x_{k},y_{k+1}))-\Psi (T x_{k+1},g(x_{k},y_{k+1}))|^p\le 2^p||\Psi ||_\infty ^p. \end{aligned}$$

Consequently,

$$\begin{aligned} {\hat{V}}_p(\Psi \circ F)^p&\le 2^{p-1}\left( H_{\alpha ,s}(\Psi )^p \Vert g\Vert _{C^1}^{\alpha p} +2{\hat{V}}_p(\Psi )^p+ 2^p||\Psi ||_\infty ^p\right) \\&\le M^p||\Psi ||_{\alpha ,s}^p+2^p{\hat{V}}_p(\Psi )^p\le (M||\Psi ||_{\alpha ,s}+2{\hat{V}}_p(\Psi ))^p. \end{aligned}$$

Therefore,

$$\begin{aligned} {\hat{V}}_p(\Psi \circ F)\le M||\Psi ||_{\alpha ,s}+2{\hat{V}}_p(\Psi ). \end{aligned}$$

Using the last inequality repeatedly and the fact that $||\Psi \circ F||_{\alpha ,s}\le ||\Psi ||_{\alpha ,s}$, we obtain the result. $\square$

For $\Psi {:}\Sigma \rightarrow {\mathbb {R}}$ piecewise $C^\alpha$, we define

$$\begin{aligned} \Vert \Psi \Vert _\alpha =\Vert \Psi \Vert _\infty +H_\alpha (\Psi ), \qquad H_{\alpha }(\Psi )=\sup _{\xi _1\ne \xi _2}\frac{|\Psi (\xi _2)-\Psi (\xi _1)|}{|\xi _2-\xi _1|^\alpha }, \end{aligned}$$

where we restrict to the cases $\xi _1,\xi _2\in \Sigma ^+$ and $\xi _1,\xi _2\in \Sigma ^-$ in the supremum.

#### Corollary 5.5

There exist $C>0$ and $\theta \in (0,1)$ such that

$$\begin{aligned} \begin{aligned}&\left| \int \Psi \cdot (\Psi \circ F_\varepsilon ^n)\, \mathrm{d}\mu _{F_\varepsilon }-\Big (\int \Psi \, \mathrm{d}\mu _{F_\varepsilon }\Big )^2\right|&\le C ||\Psi ||^2_\alpha \, \theta ^n \end{aligned} \end{aligned}$$

for all $n\ge 1$, $\varepsilon \ge 0$ and all piecewise $C^\alpha$ observables $\Psi {:}\Sigma \rightarrow {\mathbb {R}}$.

#### Proof

By Lemma 5.3,

$$\begin{aligned} ||\Pi (\Psi \circ F_\varepsilon ^j)||_{1,1/p}+{\hat{V}}_p(\Psi \circ F_\varepsilon ^j) \le \Vert \Psi \Vert _\infty +(2^{1/p}+1){\hat{V}}_p(\Psi \circ F_\varepsilon ^j). \end{aligned}$$

By Lemma 5.4, there is a constant $K_0>1$ such that

$$\begin{aligned} ||\Pi (\Psi \circ F_\varepsilon ^j)||_{1,1/p}+{\hat{V}}_p(\Psi \circ F_\varepsilon ^j) \le K_02^j(\Vert \Psi \Vert _{\alpha ,s}+{\hat{V}}_p(\Psi )). \end{aligned}$$

Hence, assumption (6) holds with $K=2K_0$. The result follows from Theorem 5.2 since $D_\Psi \le 3\Vert \Psi \Vert _\alpha$ and $\Vert \Psi \Vert _{\alpha ,s}\le \Vert \Psi \Vert _\alpha$. $\square$

### Variance Continuity for the Family of Two-Dimensional Maps

We continue to fix $\alpha \in (0,1]$.

#### Theorem 5.6

Let $\Psi _\varepsilon$, $\varepsilon \ge 0$, be piecewise $C^\alpha$ with $\sup _\varepsilon ||\Psi _\varepsilon ||_{\alpha }<\infty$. Assume that

7 $$\begin{aligned} \lim _{\varepsilon \rightarrow 0}\int |\Psi _\varepsilon -\Psi _0|\,\mathrm{d}\mu _{F_\varepsilon }=0. \end{aligned}$$

Then, $$\lim _{\varepsilon \rightarrow 0}\sigma _{F_\varepsilon }^2(\Psi _\varepsilon )=\sigma _{F_0}^2(\Psi _0).$$

(The hypotheses of this result will be verified in Sect. 6. In particular, condition (7) is addressed in Lemma 6.2.)

We first prove a lemma that will be used in the proof of Theorem 5.6.

#### Lemma 5.7

Let $\Psi {:}\Sigma \rightarrow {\mathbb {R}}$ be piecewise continuous and fix $n\ge 0$. Then,

$$\begin{aligned} \lim _{\varepsilon \rightarrow 0}\int |\Psi \circ F_0^n-\Psi \circ F_0^{n-1}\circ F_\varepsilon |\,\mathrm{d}\mu _{F_\varepsilon }=0. \end{aligned}$$

#### Proof

Let $\delta >0$, $j\ge 0$, and define $E_{\delta ,j}=\bigcup _{i=0}^j F_0^{-i}(B_\delta )$ where $B_\delta$ is the $\delta$-neighbourhood of $\Gamma$. Then, $\mu _{F_0}(E_{\delta ,n})\le (n+1)\mu _{F_0}(B_\delta )\le M\delta$ where $M=2(n+1)\Vert h_0\Vert _\infty$.

The closure of $E_{\delta ,n}$ lies in the interior of $E_{2\delta ,n}$, so there exists a continuous function $\chi {:}\Sigma \rightarrow [0,1]$ supported in $E_{2\delta ,n}$ and equal to 1 on $E_{\delta ,n}$. By statistical stability, for $\varepsilon$ sufficiently small, $\mu _{F_\varepsilon }(E_{\delta ,n}) \le \int \chi \mathrm{d}\mu _{F_\varepsilon }\le 2\int \chi \mathrm{d}\mu _{F_0}\le 2\mu _{F_0}(E_{2\delta ,n})\le 4M\delta$.

Let $E_{\delta ,j}^c=\Sigma {\setminus } E_{\delta ,j}$. Now $F_0(\xi )\in E_{\delta ,n-1}^c$ for $\xi \in E_{\delta ,n}^c$. Also, $F_\varepsilon \rightarrow F_0$ uniformly on $E_{\delta ,n}^c$ as $\varepsilon \rightarrow 0$, so $F_0(\xi ),\,F_\varepsilon (\xi )\in E_{\delta /2,n-1}^c$ for all $\xi \in E_{\delta ,n}^c$ and all sufficiently small $\varepsilon$. Moreover, $\Psi \circ F_0^{n-1}$ is uniformly continuous on $E_{\delta /2,n-1}^c$. It follows from this and the uniform convergence of $F_\varepsilon$ on $E_{\delta ,n}^c$ that $S_\varepsilon =\sup _{E_{\delta ,n}^c}|\Psi \circ F_0^{n-1}\circ F_\varepsilon -\Psi \circ F_0^n|\rightarrow 0$ as $\varepsilon \rightarrow 0$.

Hence,

$$\begin{aligned}&\int |\Psi \circ F_0^n-\Psi \circ F_0^{n-1}\circ F_\varepsilon |\,\mathrm{d}\mu _{F_\varepsilon } \le \int _{E_{\delta ,n}} |\Psi \circ F_0^n-\Psi \circ F_0^{n-1}\circ F_\varepsilon |\,\mathrm{d}\mu _{F_\varepsilon }\\&\quad +\, \int _{E_{\delta ,n}^c} |\Psi \circ F_0^n-\Psi \circ F_0^{n-1}\circ F_\varepsilon |\,\mathrm{d}\mu _{F_\varepsilon }\\&\le 2||\Psi ||_\infty \,\mu _{F_\varepsilon }(E_{\delta ,n})+S_\varepsilon \le 8||\Psi ||_\infty M\delta +S_\varepsilon \rightarrow 8||\Psi ||_\infty M\delta . \end{aligned}$$

The result follows since $\delta >0$ is arbitrary. $\square$

#### Proof of Theorem 5.6

We use the Green–Kubo formula (5). Recall that ${\hat{\Psi }}_\varepsilon =\Psi _\varepsilon -\int \Psi _\varepsilon \,\mathrm{d}\mu _{F_\varepsilon }$. By Corollary 5.5, the series in (5) is absolutely convergent uniformly in $\varepsilon$. Therefore, it suffices to show that $\int {\hat{\Psi }}_\varepsilon \cdot ({\hat{\Psi }}_\varepsilon \circ F^n_{\varepsilon })\, \mathrm{d}\mu _{F_\varepsilon }\rightarrow \int {\hat{\Psi }}_0 \cdot ({\hat{\Psi }}_0\circ F_0^n)\, \mathrm{d}\mu _{F_0}$ as $\varepsilon \rightarrow 0$ for each fixed $n\ge 0$. Now

$$\begin{aligned} \begin{aligned}&\int {\hat{\Psi }}_\varepsilon \cdot ({\hat{\Psi }}_\varepsilon \circ F^n_{\varepsilon })\, \mathrm{d}\mu _{F_\varepsilon } -\int {\hat{\Psi }}_0 \cdot ({\hat{\Psi }}_0\circ F_0^n)\, \mathrm{d}\mu _{F_0}\\&\quad =\int {\hat{\Psi }}_\varepsilon \cdot ({\hat{\Psi }}_\varepsilon \circ F^n_{\varepsilon })\, \mathrm{d}\mu _{F_\varepsilon }-\int {\hat{\Psi }}_0 \cdot ({\hat{\Psi }}_\varepsilon \circ F^n_\varepsilon )\, \mathrm{d}\mu _{F_\varepsilon }\\&\qquad +\, \int {\hat{\Psi }}_0 \cdot ({\hat{\Psi }}_\varepsilon \circ F^n_\varepsilon )\, \mathrm{d}\mu _{F_\varepsilon } -\int {\hat{\Psi }}_0 \cdot ({\hat{\Psi }}_0\circ F^n_\varepsilon )\, \mathrm{d}\mu _{F_\varepsilon }\\&\qquad +\, \int {\hat{\Psi }}_0 \cdot ({\hat{\Psi }}_0\circ F^n_\varepsilon )\, \mathrm{d}\mu _{F_\varepsilon }-\int {\hat{\Psi }}_0 \cdot ({\hat{\Psi }}_0\circ F_0^n)\, \mathrm{d}\mu _{F_\varepsilon }\\&\qquad +\, \int {\hat{\Psi }}_0 \cdot ({\hat{\Psi }}_0\circ F_0^n)\, \mathrm{d}\mu _{F_\varepsilon }-\int {\hat{\Psi }}_0 \cdot ({\hat{\Psi }}_0\circ F_0^n)\, \mathrm{d}\mu _{F_0}\\&\quad =(I) + (II) + (III) + (IV). \end{aligned} \end{aligned}$$

We have $|(II)|\le \Vert {\hat{\Psi }}_0\Vert _\infty \int |{\hat{\Psi }}_\varepsilon -{\hat{\Psi }}_0|\circ F_\varepsilon ^n\,\mathrm{d}\mu _{F_\varepsilon }= \Vert {\hat{\Psi }}_0\Vert _\infty \int |{\hat{\Psi }}_\varepsilon -{\hat{\Psi }}_0|\,\mathrm{d}\mu _{F_\varepsilon }\rightarrow 0$ by (7), and similarly $(I)\rightarrow 0$. Also, $(IV)\rightarrow 0$ by Proposition 5.1.

Finally, $|(III)|\le \Vert {\hat{\Psi }}_0\Vert _\infty \int |{\hat{\Psi }}_0\circ F_0^n-{\hat{\Psi }}_0\circ F_\varepsilon ^n|\,\mathrm{d}\mu _{F_\varepsilon }$. Note that

$$\begin{aligned} \begin{aligned}&\int |{\hat{\Psi }}_0\circ F_0^n-{\hat{\Psi }}_0\circ F_\varepsilon ^n|\,\mathrm{d}\mu _{F_\varepsilon }\\&\quad \le \sum _{i=1}^{n}\int |{\hat{\Psi }}_0\circ F_0^i\circ F_\varepsilon ^{n-i} -{\hat{\Psi }}_0\circ F_0^{i-1}\circ F_\varepsilon ^{n-i+1}|\,\mathrm{d}\mu _{F_\varepsilon }\\&\quad = \sum _{i=1}^{n}\int |{\hat{\Psi }}_0\circ F_0^i-{\hat{\Psi }}_0\circ F_0^{i-1}\circ F_\varepsilon |\,\mathrm{d}\mu _{F_\varepsilon }. \end{aligned} \end{aligned}$$

Thus, by Lemma 5.7, $(III)\rightarrow 0$ as $\varepsilon \rightarrow 0$. $\square$

We end this section with the following result which gives explicit estimates in terms of $\Psi$ as required for the proof of Theorem 1.1(b).

#### Proposition 5.8

Let $\Vert \Psi \Vert _{1,\varepsilon }= \int |\Psi |\,\mathrm{d}\mu _{F_\varepsilon }$. For all $H>0$ and $\alpha >0$, there exists $C>0$ such that

$$\begin{aligned} |\sigma _{F_\varepsilon }^2(\Psi )-\sigma _{F_\varepsilon }^2(\Psi ')|\le C \Vert \Psi -\Psi '\Vert _{1,\varepsilon }(1+ |\log \Vert \Psi -\Psi '\Vert _{1,\varepsilon }|) \end{aligned}$$

for all $\Psi$, $\Psi '$ piecewise Hölder with $\Vert \Psi \Vert _\alpha ,\,\Vert \Psi '\Vert _\alpha \le H$ and all $\varepsilon \ge 0$.

#### Proof

Let ${\hat{\Psi }}=\Psi -\int \Psi \,\mathrm{d}\mu _{F_\varepsilon }$ and ${\hat{\Psi }}'=\Psi '-\int \Psi '\,\mathrm{d}\mu _{F_\varepsilon }$. Let $N\ge 1$. It follows from the Green–Kubo formula (5) that

$$\begin{aligned} \begin{aligned}&|\sigma _{F_\varepsilon }^2(\Psi )-\sigma _{F_\varepsilon }^2(\Psi ')|\le 2\sum _{n=0}^{N-1}\Vert {\hat{\Psi }}({\hat{\Psi }}\circ F_\varepsilon ^n) -{\hat{\Psi }}'({\hat{\Psi }}'\circ F_\varepsilon ^n)\Vert _{1,\varepsilon }\\&\qquad +\, 2\sum _{n=N}^{\infty }\Vert {\hat{\Psi }}({\hat{\Psi }}\circ F_\varepsilon ^n)\Vert _{1,\varepsilon } +2\sum _{n=N}^{\infty }\Vert {\hat{\Psi }}'({\hat{\Psi }}'\circ F_\varepsilon ^n)\Vert _{1,\varepsilon }. \end{aligned} \end{aligned}$$

Now

$$\begin{aligned} \begin{aligned}&\Vert {\hat{\Psi }}({\hat{\Psi }}\circ F_\varepsilon ^n) -{\hat{\Psi }}'({\hat{\Psi }}'\circ F_\varepsilon ^n)\Vert _{1,\varepsilon } \le \Vert {\hat{\Psi }}-{\hat{\Psi }}'\Vert _{1,\varepsilon }\Vert {\hat{\Psi }}\circ F_\varepsilon ^n\Vert _\infty \\&\qquad +\, \Vert {\hat{\Psi }}'\Vert _\infty \Vert {\hat{\Psi }}\circ F_\varepsilon ^n-{\hat{\Psi }}'\circ F_\varepsilon ^n\Vert _{1,\varepsilon }\\&\quad = (\Vert {\hat{\Psi }}\Vert _\infty +\Vert {\hat{\Psi }}'\Vert _\infty )\Vert {\hat{\Psi }}-{\hat{\Psi }}'\Vert _{1,\varepsilon } \le 4(\Vert \Psi \Vert _\infty +\Vert \Psi '\Vert _\infty )\Vert \Psi -\Psi '\Vert _{1,\varepsilon }. \end{aligned} \end{aligned}$$

Also, by Corollary 5.5, $\Vert {\hat{\Psi }}({\hat{\Psi }}\circ F_\varepsilon ^n)\Vert _{1,\varepsilon }\le C\theta ^n\Vert {\hat{\Psi }}\Vert _\alpha ^2 \le 4C\theta ^n\Vert \Psi \Vert _\alpha ^2$ and similarly for $\Psi '$. Hence,

$$\begin{aligned} |\sigma _{F_\varepsilon }^2(\Psi )-\sigma _{F_\varepsilon }^2(\Psi ')|\le 8N(\Vert \Psi \Vert _\infty +\Vert \Psi '\Vert _\infty )\Vert \Psi -\Psi '\Vert _{1,\varepsilon } +C'\theta ^N(\Vert \Psi \Vert _\alpha ^2+\Vert \Psi '\Vert _\alpha ^2). \end{aligned}$$

where $C'=8C(1-\theta )^{-1}$. Taking $N=[q|\log \Vert \Psi -\Psi '\Vert _{1,\varepsilon }|]$ for *q* sufficiently large yields the desired result. $\square$

## Variance Continuity for the Flows

By [5, 16], the CLT holds for Hölder observables for the Lorenz flows $X_\varepsilon$. In this section, we show how to obtain continuity of the variances, proving Theorem 1.1. During most of this section, we continue to work with normalised families as in Sect. 3, culminating in Corollary 6.5 which is an analogue of Theorem 1.1 for normalised families. We conclude by using Corollary 6.5 to prove Theorem 1.1. Throughout, we fix $\beta \in (0,1)$.

First, we recall some results from the literature. Let $\psi {:}{\mathbb {R}}^3\rightarrow {\mathbb {R}}$ be $C^\beta$. Define

$$\begin{aligned} {{\tilde{\psi }}}(x)=\psi (x)-\psi (0). \end{aligned}$$

Also, define the induced observables $\Psi _{\varepsilon }{:}\Sigma {\setminus }\Gamma \rightarrow {\mathbb {R}}$, $\varepsilon \ge 0$, by

8 $$\begin{aligned} \Psi _{\varepsilon }(\xi )=\int _{0}^{\tau _{\varepsilon }(\xi )}{{\tilde{\psi }}}(X_\varepsilon (\xi , t))\,\mathrm{d}t. \end{aligned}$$

The left-hand side of (2) is identical for $\psi$ and ${{\tilde{\psi }}}$, so

9 $$\begin{aligned} \sigma ^2_{X_\varepsilon }(\psi )=\sigma ^2_{X_\varepsilon }({{\tilde{\psi }}}). \end{aligned}$$

### Proposition 6.1

The variances for ${{\tilde{\psi }}}$ and $\Psi _{\varepsilon }$ are related by

$$\begin{aligned} \sigma ^2_{X_\varepsilon }({{\tilde{\psi }}})=\frac{\sigma _{F_\varepsilon }^2( \Psi _{\varepsilon })}{\int \tau _\varepsilon \, \mathrm{d}\mu _{F_\varepsilon }}. \end{aligned}$$

### Proof

By [16], ${{\tilde{\psi }}}$ satisfies the CLT. The method of proof in [16] is to show that $\Psi _{\varepsilon }$ and $\tau _\varepsilon$ satisfy the CLT, whereupon the CLT for ${{\tilde{\psi }}}$ follows from [22, Theorem 1.1]. The desired relation between the variances is given explicitly in [22, Theorem 1.1]. $\square$

By [2, Lemma 4.1],

10 $$\begin{aligned} \lim _{\varepsilon \rightarrow 0}\int \tau _\varepsilon \, \mathrm{d}\mu _{F_\varepsilon }=\int \tau _0\, \mathrm{d}\mu _{F_0}. \end{aligned}$$

Hence, the main quantity to control is $\sigma _{F_\varepsilon }^2( \Psi _{\varepsilon })$. First, we verify condition (7) in Theorem 5.6.

### Lemma 6.2

$$\lim _{\varepsilon \rightarrow 0}\int |\Psi _{\varepsilon }-\Psi _{0}|\,\mathrm{d}\mu _{F_\varepsilon }=0$$.

### Proof

Fix $N\ge 1$ and set $\tau _{\varepsilon , N}(\xi )=\min \{\tau _{ \varepsilon }(\xi ), N\}$. Define

$$\begin{aligned} \Psi _{\varepsilon , N}(\xi )=\int _{0}^{\tau _{\varepsilon , N}(\xi )}{{\tilde{\psi }}}(X_\varepsilon (\xi , t))\,\mathrm{d}t. \end{aligned}$$

Then,

$$\begin{aligned} |\Psi _0-\Psi _{\varepsilon }|\le |\Psi _0-\Psi _{0,N}|+|\Psi _{0,N}- \Psi _{\varepsilon , N}|+|\Psi _{\varepsilon , N}-\Psi _{\varepsilon }|. \end{aligned}$$

Now,

$$\begin{aligned} |\Psi _\varepsilon (\xi )-\Psi _{\varepsilon , N}(\xi )|=\Big |\int _{\tau _{\varepsilon , N}(\xi )}^{\tau _{\varepsilon }(\xi )}{{\tilde{\psi }}}(X_\varepsilon (\xi , t))\,\mathrm{d}t\Big | \le 2\Vert \psi \Vert _{\infty }(\tau _{\varepsilon }(\xi )-\tau _{\varepsilon , N}(\xi )). \end{aligned}$$

Recall that $\xi =(x,y,1)$. Now $\tau _\varepsilon (\xi ) -\tau _{\varepsilon ,N}(\xi )\le -C\log |x|-N$, and $\tau _\varepsilon -\tau _{\varepsilon ,N}$ is supported on $B_\varepsilon =\{\xi {:}\tau _\varepsilon (\xi ) >N\}\subset \{\xi {:} |x|\ <e^{-N/C}\}$. Hence,

$$\begin{aligned} \begin{aligned} \int&|\Psi _\varepsilon -\Psi _{\varepsilon , N}|\,\mathrm{d}\mu _{F_\varepsilon } \le 2\Vert h_\varepsilon \Vert _\infty \Vert \psi \Vert _\infty \int _{B_\varepsilon }(-C\log |x|-N)\,dx \\&= 4\Vert h_\varepsilon \Vert _\infty \Vert \psi \Vert _\infty \int _0^{e^{-N/C}}(-C\log x-N)\,dx =4C\Vert h_\varepsilon \Vert _\infty {||\psi ||}_{\infty } e^{-N/C}. \end{aligned} \end{aligned}$$

By Proposition 4.1(a), there exists $C'>0$ such that $\int |\Psi _\varepsilon -\Psi _{\varepsilon , N}|\,\mathrm{d}\mu _{F_\varepsilon } \le C'e^{-N/C}\Vert \psi \Vert _\infty$. Similarly, $\int |\Psi _0-\Psi _{0, N}|\,\mathrm{d}\mu _{F_\varepsilon } \le C'e^{-N/C}\Vert \psi \Vert _\infty$.

Next, $$|\Psi _{\varepsilon ,N}(\xi )-\Psi _{0,N}(\xi )| \le (I)+(II)$$ where

$$\begin{aligned} \begin{aligned} (I) =&\int _{\tau _{0,N}(\xi )}^{\tau _{\varepsilon ,N}(\xi )}|{{\tilde{\psi }}}(X_\varepsilon (\xi ,t))|\,\mathrm{d}t \le 2\Vert \psi \Vert _\infty |\tau _{\varepsilon ,N}(\xi )-\tau _{0,N}(\xi )|, \\ (II) =&\int _0^{\tau _{0,N}(\xi )}|{{\tilde{\psi }}}(X_\varepsilon (\xi ,t))-{{\tilde{\psi }}}(X_0(\xi ,t))|\,\mathrm{d}t\\ \le&\int _0^N|\psi (X_\varepsilon (\xi ,t))-\psi (X_0(\xi ,t))|\,\mathrm{d}t \le H_\beta (\psi )\int _0^N|X_\varepsilon (\xi ,t)-X_0(\xi ,t)|^\beta \,\mathrm{d}t. \end{aligned} \end{aligned}$$

For $\delta >0$ fixed sufficiently small, there exists $\varepsilon _0>0$ such that $\tau _{\varepsilon ,N}(\xi )\equiv N$ for $|x|<\delta$ and $\varepsilon <\varepsilon _0$. Also, it follows from smoothness of the flow and boundedness of first hit times for $|x|\ge \delta$ that $\tau _{\varepsilon ,N}\rightarrow \tau _{0,N}$ uniformly on $\{|x|\ge \delta \}$. Hence, $\lim _{\varepsilon \rightarrow 0}\Vert \tau _{\varepsilon ,N}-\tau _{0,N}\Vert _\infty =0$ and so $\lim _{\varepsilon \rightarrow 0}\Vert (I)\Vert _\infty =0$.

By continuity of the flow in initial conditions and parameters, $X_\varepsilon (\xi ,t)\rightarrow X_0(\xi ,t)$ uniformly in $\xi \in \Sigma$ and $t\in [0,N]$. Hence, $\lim _{\varepsilon \rightarrow 0}\Vert (II)\Vert _\infty =0$.

We have shown that $\mathop {{\overline{{\mathrm{lim}}}}}_{\varepsilon \rightarrow 0}\int |\Psi _\varepsilon -\Psi _0|\,\mathrm{d}\mu _{F_\varepsilon } \le 2C'e^{-N/C}$. The result follows since *N* is arbitrary. $\square$

Next, we show that $\sup _\varepsilon \Vert \Psi _\varepsilon \Vert _\alpha <\infty$ for some $\alpha >0$.

### Lemma 6.3

Define ${\widetilde{\Psi }}_\varepsilon {:}\Sigma \rightarrow {\mathbb {R}}$ by setting

$$\begin{aligned} {\widetilde{\Psi }}_\varepsilon (x,y)=\int _0^{-\frac{1}{\lambda _{1,\varepsilon }}\log |x|} {{\tilde{\psi }}}(xe^{\lambda _{1,\varepsilon }t},ye^{\lambda _{2,\varepsilon }t},e^{\lambda _{3,\varepsilon }t})\,\mathrm{d}t. \end{aligned}$$

Choose $0<\alpha '<-\lambda _{3,\varepsilon }\beta /(\lambda _{1,\varepsilon }-\lambda _{3,\varepsilon })$. Then, ${\widetilde{\Psi }}_\varepsilon$ is piecewise $C^{\alpha '}$. Moreover, there is a constant $C>0$ such that $\Vert {\widetilde{\Psi }}_\varepsilon \Vert _{\alpha '}\le C\Vert \psi \Vert _\beta$ for all $\varepsilon \ge 0$.

### Proof

We suppress the dependence on $\varepsilon$. Write ${{\tilde{\psi }}}(x,y,z)=\psi _1(x)+\psi _2(x,y,z)$ where $\psi _1(x)={{\tilde{\psi }}}(x,0,0)$. Then, $\psi _1$ and $\psi _2$ are Hölder with $H_\beta (\psi _1)\le H_\beta (\psi )$ and $H_\beta (\psi _2)\le 2H_\beta (\psi )$. Also, $\psi _1(0)=0$ and $\psi _2(x,0,0)\equiv 0$. Define

$$\begin{aligned} {\widetilde{\Psi }}_1(x)= & {} \int _0^{-\frac{1}{\lambda _1}\log x} \psi _1(xe^{\lambda _1t})\,\mathrm{d}t, \quad {\widetilde{\Psi }}_2(x,y)\\= & {} \int _0^{-\frac{1}{\lambda _1}\log x} \psi _2(xe^{\lambda _1t},ye^{\lambda _2t},e^{\lambda _3t})\,\mathrm{d}t. \end{aligned}$$

Recall that $$\lambda _2<\lambda _3<0<\lambda _1$$.

First, we carry out the estimates for ${\widetilde{\Psi }}_1$ with $\alpha '=\beta$. By the change of variables $u=xe^{\lambda _1t}$,

$$\begin{aligned} {\widetilde{\Psi }}_1(x)=\frac{1}{\lambda _1}\int _x^1 \frac{\psi _1(u)}{u}\,du. \end{aligned}$$

Now $|\psi _1(u)|=|\psi _1(u)-\psi _1(0)|\le H_\beta (\psi ) u^\beta$, so $|{\widetilde{\Psi }}_1|_\infty \le \frac{H_\beta (\psi )}{\lambda _1} \int _0^1 u^{-(1-\beta )}\,du =\frac{H_\beta (\psi )}{\lambda _1\beta }$. Also, for $x_1>x_2>0$,

$$\begin{aligned} \begin{aligned} |{\widetilde{\Psi }}_1(x_1)-{\widetilde{\Psi }}_1(x_2)|&\le \frac{H_\beta (\psi )}{\lambda _1} \int _{x_2}^{x_1}u^{-(1-\beta )}\,du \\&=\frac{H_\beta (\psi )}{\lambda _1\beta } (x_1^\beta -x_2^\beta ) \le \frac{H_\beta (\psi )}{\lambda _1\beta } (x_1-x_2)^\beta . \end{aligned} \end{aligned}$$

Here, we have used that $x_1^\beta -x_2^\beta \le (x_1-x_2)^\beta$ for all $\beta \in [0,1]$. Hence, $\Vert {\widetilde{\Psi }}_1\Vert _\beta \le \frac{1}{\lambda _1\beta }\Vert \psi \Vert _\beta$.

Next, we carry out the estimates for ${\widetilde{\Psi }}_2$. Note that

11 $$\begin{aligned} \ |\psi _2(xe^{\lambda _1t},ye^{\lambda _2t},e^{\lambda _3t})|&= |\psi _2(xe^{\lambda _1t},ye^{\lambda _2t},e^{\lambda _3t})-\psi _2(xe^{\lambda _1 t},0,0)|\nonumber \\&= |\psi _2(xe^{\lambda _1t},ye^{\lambda _2t},e^{\lambda _3t}) -\psi _2(xe^{\lambda _1t},ye^{\lambda _2t},0)| \nonumber \\&\quad +\, |\psi _2(xe^{\lambda _1t},ye^{\lambda _2t},0)-\psi _2(xe^{\lambda _1t},0,0)| \nonumber \\&\le H_\beta (\psi _2)(e^{\beta \lambda _3 t}+|y|^\beta e^{\beta \lambda _2 t})\le 4 H_\beta (\psi ) e^{\beta \lambda _3 t}. \end{aligned}$$

In particular,

$$\begin{aligned} |{\widetilde{\Psi }}_2|_\infty \le 4H_\beta (\psi ) \int _0^{-\frac{1}{\lambda _1}\log x} e^{\beta \lambda _3 t}\,\mathrm{d}t =\frac{4H_\beta (\psi )}{\beta |\lambda _3|} (1-x^{-\beta \lambda _3/\lambda _1})\le \frac{4H_\beta (\psi )}{\beta |\lambda _3|}. \end{aligned}$$

Similarly,

$$\begin{aligned}&|{\widetilde{\Psi }}_2(x,y_1)-{\widetilde{\Psi }}_2(x,y_2)|\\&\quad \le \int _0^{-\frac{1}{\lambda _1}\log x}H_\beta (\psi _2)|y_1-y_2|^\beta e^{\beta \lambda _2t}\,\mathrm{d}t\le \frac{2H_\beta (\psi )}{\beta |\lambda _2|} |y_1-y_2|^\beta . \end{aligned}$$

For $x_1>x_2>0$, we have $|{\widetilde{\Psi }}_2(x_1,y)-{\widetilde{\Psi }}_2(x_2,y)|\le A+B$ where

$$\begin{aligned} A&= \int _{-\frac{1}{\lambda _1}\log x_1}^{-\frac{1}{\lambda _1}\log x_2} \psi _2(x_2e^{\lambda _1t},ye^{\lambda _2t},e^{\lambda _3t})\,\mathrm{d}t, \\ B&= \int _0^{-\frac{1}{\lambda _1}\log x_1}|\psi _2(x_1e^{\lambda _1t}, ye^{\lambda _2t},e^{\lambda _3t})-\psi _2(x_2e^{\lambda _1t}, ye^{\lambda _2t},e^{\lambda _3t})|\,\mathrm{d}t. \end{aligned}$$

By (11),

$$\begin{aligned} A\le 4 H_\beta (\psi ) \int _{-\frac{1}{\lambda _1}\log x_1}^{-\frac{1}{\lambda _1}\log x_2} e^{\beta \lambda _3t}\,\mathrm{d}t =\frac{4H_\beta (\psi )}{\beta |\lambda _3|}(x_1^{\gamma }-x_2^{\gamma })\le \frac{4H_\beta (\psi )}{\beta |\lambda _3|} (x_1-x_2)^{\gamma } \end{aligned}$$

where $$\gamma =-\lambda _3\beta /\lambda _1$$. Let

$$\begin{aligned} \alpha '=\delta \beta , \qquad \lambda '=\delta \lambda _1+(1-\delta )\lambda _3, \end{aligned}$$

where $0<\delta<-\lambda _3/(\lambda _1-\lambda _3)<1$. In particular, $\alpha '<\gamma$ and $\lambda '<0$. Since the eigenvalues $\lambda _1,\lambda _3$ depend continuously on $\varepsilon$, we can choose $\delta$ independent of $\varepsilon$. The inequality $\min \{a,b\}\le a^\delta b^{1-\delta }$ holds for all $a,b\ge 0$. By (11),

$$\begin{aligned}&|\psi _2(x_1e^{\lambda _1t},ye^{\lambda _2t},e^{\lambda _3t}) -\psi _2(x_2e^{\lambda _1t},ye^{\lambda _2t},e^{\lambda _3t})|\\&\quad \le \min \{H_\beta (\psi _2)(x_1-x_2)^\beta e^{\beta \lambda _1 t}, 8H_\beta (\psi ) e^{\beta \lambda _3 t}\} \le 8H_\beta (\psi ) (x_1-x_2)^{\delta \beta } e^{\delta \lambda ' t}. \end{aligned}$$

Hence, $B\le \frac{8}{\delta |\lambda '|} H_\beta (\psi ) (x_1-x_2)^{\alpha '}$ completing the proof. $\square$

### Corollary 6.4

There exists $\alpha \in (0,\beta )$ such that $\Psi _\varepsilon$ is piecewise $C^{\alpha }$. Moreover, there is a constant $C>0$ such that $\sup _\varepsilon \Vert \Psi _\varepsilon \Vert _{\alpha }\le C\Vert \psi \Vert _\beta$.

### Proof

After a $C^\alpha$ change of coordinates in a neighbourhood of the singularity, we may suppose without loss of generality that the flow $X_\varepsilon$ is linear near the singularity. Here, $\alpha >0$ can be chosen independent of $\varepsilon$ with bounded Hölder constants for the linearisation [10]. We choose $\alpha <\alpha '$ where $\alpha '$ is as in Lemma 6.3

For the remainder of the proof, we suppress the dependence on $\varepsilon$. The first hit time in this neighbourhood is given by $\tau _1(\xi )=-\frac{1}{\lambda _1}\log |x|$. Recall that $\xi =(x,y,1)$ and set $\xi '=X(\xi ,\tau _1(\xi ))=(1,yx^{-\lambda _2/\lambda _1},x^{-\lambda _3/\lambda _1})$. Note that the dependence of $\xi '$ on $\xi$ is Hölder (also uniformly in $\varepsilon$). Then, $\tau =\tau _1+\tau _2$ where $\tau _2$ is $C^\alpha$ in $\xi '$ and hence $\xi$ (uniformly in $\varepsilon$). Also, $\Psi ={\widetilde{\Psi }}+{\widehat{\Psi }}$ where

12 $$\begin{aligned} {\widetilde{\Psi }}(\xi )=\int _0^{\tau _1(\xi )}{{\tilde{\psi }}}(xe^{\lambda _1 t},ye^{\lambda _2 t},e^{\lambda _3 t})\,\mathrm{d}t, \quad {\widehat{\Psi }}(\xi )=\int _0^{\tau _2(\xi )}{{\tilde{\psi }}}(X(\xi ',t))\,\mathrm{d}t. \end{aligned}$$

Since $\Vert \tau _2\Vert _\infty$ is bounded, it is immediate that ${\widehat{\Psi }}$ is $C^\alpha$ uniformly in $\varepsilon$. By Lemma 6.3, the same is true for ${\widetilde{\Psi }}$. $\square$

We can now state and prove the analogue of Theorem 1.1 for normalised families. Define $\Vert \psi \Vert _{(\varepsilon )}=\int |\psi |\,\mathrm{d}\mu _\varepsilon +|\psi (0)|$.

### Corollary 6.5

Let $X_\varepsilon$ be a normalised family of flows admitting geometric Lorenz attractors. Then,

- $\lim _{\varepsilon \rightarrow 0}\sigma ^2_{X_\varepsilon }(\psi )=\sigma ^2_{X_0}(\psi )$ for all $C^\beta$ observables $\psi {:}{\mathbb {R}}^3\rightarrow {\mathbb {R}}$
- For any $H>0$, there exists $C>0$ such that
  $$\begin{aligned} |\sigma ^2_{X_\varepsilon }(\psi )-\sigma ^2_{X_\varepsilon }(\psi ')|\le C\Vert \psi -\psi '\Vert _{(\varepsilon )}(1+|\log \Vert \psi -\psi '\Vert _{(\varepsilon )}|) \end{aligned}$$
  for all $C^\beta$ observables $\psi ,\,\psi '{:}{\mathbb {R}}^3\rightarrow {\mathbb {R}}$ with $\Vert \psi \Vert _\beta ,\,\Vert \psi '\Vert _\beta \le H$, and all $\varepsilon \ge 0$.

### Proof

By Corollary 6.4, there exists $C_1>0$ and $\alpha \in (0,1)$ such that $\Psi _\varepsilon$, $\Psi '_\varepsilon$ are piecewise $C^\alpha$ and

13 $$\begin{aligned} \sup _\varepsilon \Vert \Psi _\varepsilon \Vert _\alpha \le C_1\Vert \psi \Vert _\beta , \qquad \sup _\varepsilon \Vert \Psi '_\varepsilon \Vert _\alpha \le C_1\Vert \psi '\Vert _\beta . \end{aligned}$$

By (9) and Proposition 6.1,

$$\begin{aligned} \begin{aligned}&|\sigma ^2_{X_\varepsilon }(\psi )-\sigma ^2_{X_{\varepsilon '}}(\psi ')| =|\sigma ^2_{X_\varepsilon }({{\tilde{\psi }}})-\sigma ^2_{X_{\varepsilon '}}({{\tilde{\psi }}}')| =\left| \frac{\sigma _{F_\varepsilon }^2( \Psi _\varepsilon )}{\int \tau _\varepsilon \, \mathrm{d}\mu _{F_\varepsilon }}- \frac{\sigma _{F_{\varepsilon '}}^2 ( \Psi '_{\varepsilon '})}{\int \tau _{\varepsilon '}\, \mathrm{d}\mu _{F_{\varepsilon '}}}\right| \\&\le \sigma _{F_{\varepsilon '}}^2( \Psi '_{\varepsilon '}) \left| \frac{1}{\int \tau _\varepsilon \, \mathrm{d}\mu _{F_\varepsilon }}- \frac{1}{\int \tau _{\varepsilon '}\, \mathrm{d}\mu _{F_{\varepsilon '}}}\right| +\frac{1}{\int \tau _\varepsilon \, \mathrm{d}\mu _{F_\varepsilon }} \left| \sigma _{F_\varepsilon }^2( \Psi _{\varepsilon })- \sigma _{F_{\varepsilon '}}^2( \Psi '_{\varepsilon '}) \right| . \end{aligned}\end{aligned}$$

First suppose that $\psi =\psi '$ and $\varepsilon '=0$. By Lemma 6.2 and (13), we can apply Theorem 5.6 to deduce that $\lim _{\varepsilon \rightarrow 0}\sigma _{F_\varepsilon }^2( \Psi _\varepsilon )= \sigma _{F_0}^2( \Psi _0)$. Part (a) now follows from (10).

Next suppose that $\varepsilon =\varepsilon '$. By Proposition 5.8 and (13), there is a constant $C>0$ (independent of $\varepsilon$) such that $| \sigma _{F_\varepsilon }^2( \Psi _\varepsilon )- \sigma _{F_\varepsilon }^2( \Psi '_\varepsilon ) |\le C \Vert \Psi _\varepsilon -\Psi '_\varepsilon \Vert _{1,\varepsilon } (1+|\log \Vert \Psi _\varepsilon -\Psi '_\varepsilon \Vert _{1,\varepsilon }|)$ where

$$\begin{aligned} \begin{aligned} \Vert \Psi _\varepsilon -\Psi '_\varepsilon \Vert _{1,\varepsilon }&=\int |\Psi _\varepsilon -\Psi '_\varepsilon |\,\mathrm{d}\mu _{F_\varepsilon }\\&\le \int \int _0^{\tau _\varepsilon (\xi )}|{{\tilde{\psi }}}(X_\varepsilon (\xi ,t) -{{\tilde{\psi }}}'(X_\varepsilon (\xi ,t)|\,\mathrm{d}t\,\mathrm{d}\mu _{F_\varepsilon }\\&= \int \tau _\varepsilon \, \mathrm{d}\mu _{F_\varepsilon }\int |{{\tilde{\psi }}}-{{\tilde{\psi }}}'|\,\mathrm{d}\mu _\varepsilon \\&\le \int \tau _\varepsilon \, \mathrm{d}\mu _{F_\varepsilon }\Big (\int |\psi -\psi '|\,\mathrm{d}\mu _\varepsilon +|\psi (0)-\psi '(0)|\Big ). \end{aligned}\end{aligned}$$

This proves part (b). $\square$

### Proof of Theorem 1.1

Let $\phi _\varepsilon {:}U\rightarrow U$ be the family of normalising conjugacies in Sect. 3. Recall that $\tilde{X}_\varepsilon (x,t)=\phi _\varepsilon ^{-1}\circ X_\varepsilon (\phi _\varepsilon (x),t)$. Define

$$\begin{aligned} \psi _\varepsilon =\psi \circ \phi _\varepsilon , \quad \psi '_\varepsilon =\psi '\circ \phi _\varepsilon , \quad {{\tilde{\mu }}}_\varepsilon ={\phi _\varepsilon ^{-1}}\!_*\mu _\varepsilon . \end{aligned}$$

Then, $\psi _\varepsilon$ is Hölder and

$$\begin{aligned} \int \psi _\varepsilon \,d{{\tilde{\mu }}}_\varepsilon = \int \psi \,\mathrm{d}\mu _\varepsilon , \qquad \int _0^t \psi _\varepsilon \circ {\tilde{X}}_\varepsilon (s)\,\mathrm{d}s= \Big (\int _0^t \psi \circ X_\varepsilon (s)\,\mathrm{d}s\Big )\circ \phi _\varepsilon . \end{aligned}$$

Hence, $\int _0^t \psi \circ X_\varepsilon (s)\,\mathrm{d}s-t\int \psi \,\mathrm{d}\mu _\varepsilon$ and $\int _0^t \psi _\varepsilon \circ \tilde{X}_\varepsilon (s)\,\mathrm{d}s-t\int \psi _\varepsilon \,d{{\tilde{\mu }}}_\varepsilon$ have the same distribution (relative to the probability measures $\mu _\varepsilon$ and ${{\tilde{\mu }}}_\varepsilon$, respectively), so it follows from (2) that $\sigma ^2_{X_\varepsilon }(\psi )=\sigma ^2_{{\tilde{X}}_\varepsilon }(\psi _\varepsilon )$. Similar comments apply to $\psi '$.

It follows from the definitions that ${\tilde{X}}_\varepsilon$ is a normalised family. By Corollary 3.2, there exist $H>0$ and $\beta \in (0,1)$ such that $\sup _\varepsilon \Vert \psi _\varepsilon \Vert _\beta \le H$ and $\sup _\varepsilon \Vert \psi '_\varepsilon \Vert _\beta \le H$.

By Corollary 6.5(b), there is a constant $C>0$ such that

$$\begin{aligned} \begin{aligned} |\sigma _{X_\varepsilon }^2(\psi )- \sigma _{X_0}^2(\psi )|&=|\sigma _{\tilde{X}_\varepsilon }^2(\psi _\varepsilon )-\sigma _{{\tilde{X}}_0}^2(\psi _0)| \\&\le |\sigma _{{\tilde{X}}_\varepsilon }^2(\psi _\varepsilon )-\sigma _{{\tilde{X}}_\varepsilon }^2(\psi _0)| +|\sigma _{{\tilde{X}}_\varepsilon }^2(\psi _0)-\sigma _{{\tilde{X}}_0}^2(\psi _0)| \\&\le C\Vert \psi _\varepsilon -\psi _0\Vert _\infty (1+|\log \Vert \psi _\varepsilon -\psi _0\Vert _\infty |)\\&\quad +\, |\sigma _{{\tilde{X}}_\varepsilon }^2(\psi _0)-\sigma _{{\tilde{X}}_0}^2(\psi _0)|. \end{aligned} \end{aligned}$$

By Corollary 6.5(a), the second term on the right-hand side converges to zero as $\varepsilon \rightarrow 0$. Also, $\lim _{\varepsilon \rightarrow 0}\Vert \psi _\varepsilon -\psi _0\Vert _\infty =\lim _{\varepsilon \rightarrow 0}\Vert \psi \circ \phi _\varepsilon ^{-1}-\psi \circ \phi _0^{-1}\Vert _\infty =0$ by Corollary 3.2. Hence, $\lim _{\varepsilon \rightarrow 0} \sigma _{X_\varepsilon }^2(\psi )=\sigma _{X_0}^2(\psi )$ proving part (a).

Finally, by Corollary 6.5(b),

$$\begin{aligned} \begin{aligned} |\sigma ^2_{X_0}(\psi )-\sigma ^2_{X_0}(\psi ')&=|\sigma ^2_{\tilde{X}_0}(\psi _0)-\sigma ^2_{{\tilde{X}}_0}(\psi '_0)| \\&\le C\Vert \psi _0-\psi '_0\Vert _{(0)}(1+|\log \Vert \psi _0-\psi '_0\Vert _{(0)}|) \\&= C\Vert \psi -\psi '\Vert (1+|\log \Vert \psi -\psi '\Vert |) \end{aligned} \end{aligned}$$

yielding part (b). $$\square $$
